# Hypoxia-mediated upregulation of MCT1 expression supports the glycolytic phenotype of glioblastomas

**DOI:** 10.18632/oncotarget.10114

**Published:** 2016-06-16

**Authors:** Vera Miranda-Gonçalves, Sara Granja, Olga Martinho, Mrinalini Honavar, Marta Pojo, Bruno M. Costa, Manuel M. Pires, Célia Pinheiro, Michelle Cordeiro, Gil Bebiano, Paulo Costa, Rui M. Reis, Fátima Baltazar

**Affiliations:** ^1^ Life and Health Sciences Research Institute (ICVS), School of Health Sciences, University of Minho, Braga, Portugal; ^2^ ICVS/3B's-PT Government Associate Laboratory, Braga/Guimarães, Portugal; ^3^ Molecular Oncology Research Center, Barretos Cancer Hospital, Barretos, São Paulo, Brazil; ^4^ Department of Pathology, Hospital Pedro Hispano, Matosinhos, Portugal; ^5^ Unit of Neuropathology, Centro Hospitalar do Porto, Porto, Portugal; ^6^ Department of Neurosurgery, Centro Hospitalar do Porto, Porto, Portugal; ^7^ Hospital Dr. Nélio Mendonça, Funchal, Madeira, Portugal; ^8^ Radiotherapy Service, Centro Hospitalar do Montijo, Setúbal, Portugal

**Keywords:** monocarboxylate transporters (MCTs), tumor hypoxia, lactate, glioblastomas, Warburg effect

## Abstract

**Background:**

Glioblastomas (GBM) present a high cellular heterogeneity with conspicuous necrotic regions associated with hypoxia, which is related to tumor aggressiveness. GBM tumors exhibit high glycolytic metabolism with increased lactate production that is extruded to the tumor microenvironment through monocarboxylate transporters (MCTs). While hypoxia-mediated regulation of MCT4 has been characterized, the role of MCT1 is still controversial. Thus, we aimed to understand the role of hypoxia in the regulation of MCT expression and function in GBM, MCT1 in particular.

**Methods:**

Expression of hypoxia- and glycolytic-related markers, as well as MCT1 and MCT4 isoforms was assessed in *in vitro* and *in vivo* orthotopic glioma models, and also in human GBM tissues by immunofluorescence/immunohistochemistry and Western blot. Following MCT1 inhibition, either pharmacologically with CHC (α-cyano-4-hydroxynnamic acid) or genetically with siRNAs, we assessed GBM cell viability, proliferation, metabolism, migration and invasion, under normoxia and hypoxia conditions.

**Results:**

Hypoxia induced an increase in MCT1 plasma membrane expression in glioma cells, both in *in vitro* and *in vivo* models. Additionally, treatment with CHC and downregulation of MCT1 in glioma cells decreased lactate production, cell proliferation and invasion under hypoxia. Moreover, in the *in vivo* orthotopic model and in human GBM tissues, there was extensive co-expression of MCT1, but not MCT4, with the GBM hypoxia marker CAIX.

**Conclusion:**

Hypoxia-induced MCT1 supports GBM glycolytic phenotype, being responsible for lactate efflux and an important mediator of cell survival and aggressiveness. Therefore, MCT1 constitutes a promising therapeutic target in GBM.

## BACKGROUND

Tumor cells present several biological alterations known as cancer hallmarks [1]. Recently, metabolic reprogramming, namely “Warburg effect”, was considered a major cancer feature [2-4], in which glycolytic phenotype is favored. The glycolytic phenotype observed in tumor cells is also an adaptive consequence of tumor hypoxic microenvironment [4]. The presence of tumor hypoxia induces stabilization of hypoxia-inducible factor 1 alpha (HIF-1α) [5], which leads not only to induction of angiogenesis, but also upregulation of glycolytic metabolism, to maintain the ATP production necessary for cell survival and proliferation [6]. The increased lactate production together with diminished vascular dispersion of CO_2_ contributes to tumor hypoxic acidosis [6], which is associated with increased aggressiveness, poor prognosis and acquired resistance [7].

Glioblastoma (GBM) is the most common and lethal primary brain tumor [8]. Despite the observed progress in therapy, prognosis of GBM patients is still dismal, presenting an overall survival of approximately 15 months [9]. Thus, novel and more effective therapeutic approaches are needed to overcome this scenario. GBMs are known to exhibit metabolic remodeling [10], with an increase in glycolysis of about 3-fold, compared to normal brain tissue [11]. Additionally, GBM cells have increased lactate production [12], which is further transported to the tumor microenvironment through monocarboxylate transporters (MCTs) [13].

MCTs belong to the solute carrier family 16 (SLC16), which is composed by 14 members, being MCT1-MCT4 responsible by the proton-coupled transport of monocarboxylates [14]. The activity of these transporters is critical for metabolic communication between cells and they have different properties and tissue distributions, according to their kinetics characteristics [15]. According to the distinct affinities of MCT isoforms for lactate, MCT1 and MCT4 are described to mediate lactate efflux, contributing to tumor pH regulation [15]. Several studies from our group and others reported up-regulation of MCTs in different solid tumors [16]. Recently, we demonstrated that MCT1/4 and their chaperone CD147 are up-regulated on GBMs and presented evidence for the role of MCT1 in the hyper-glycolytic and acid-resistant phenotype of these tumors [17].

There is increasing evidence that MCT expression is altered (at transcriptional and post-transcriptional levels) in response to metabolic demands [18]. Hypoxia is a common feature of solid tumors, including GBMs, and it promotes tumor aggressiveness, leading to poor patient outcome [19]. MCT4 expression was described to be regulated by hypoxia in several cancer types, including bladder [20], cervix [21] and breast [22, 23]. Also, in normal tissues, MCT4 was described to be upregulated after chronic hypoxia, being increased in heart muscle [24], trophoblast [25] and adipocyte [26] cells in a HIF-1α dependent mechanism. In fact, following elegant functional studies, Ullah and collaborators, showed that MCT4, but not MCT1 promoter was activated by hypoxia and that this response was mediated by HIF-1α [27]. Nevertheless, it was observed that MCT1 increased in neuronal, astrocytes and endothelial cells upon left middle cerebral artery occlusion [28], as well as in adipocytes exposed to hypoxia [26]. Thus, the regulation of MCT1 under hypoxia conditions appears to be not well understood and even controversial. Therefore, in the present study we aim to understand how hypoxia regulates MCT1 activity and expression and how this associates with aggressiveness and survival of GBM cells.

## RESULTS

### Hypoxia increases MCT1 plasma membrane expression in glioma cells in vitro

**Figure 1:**
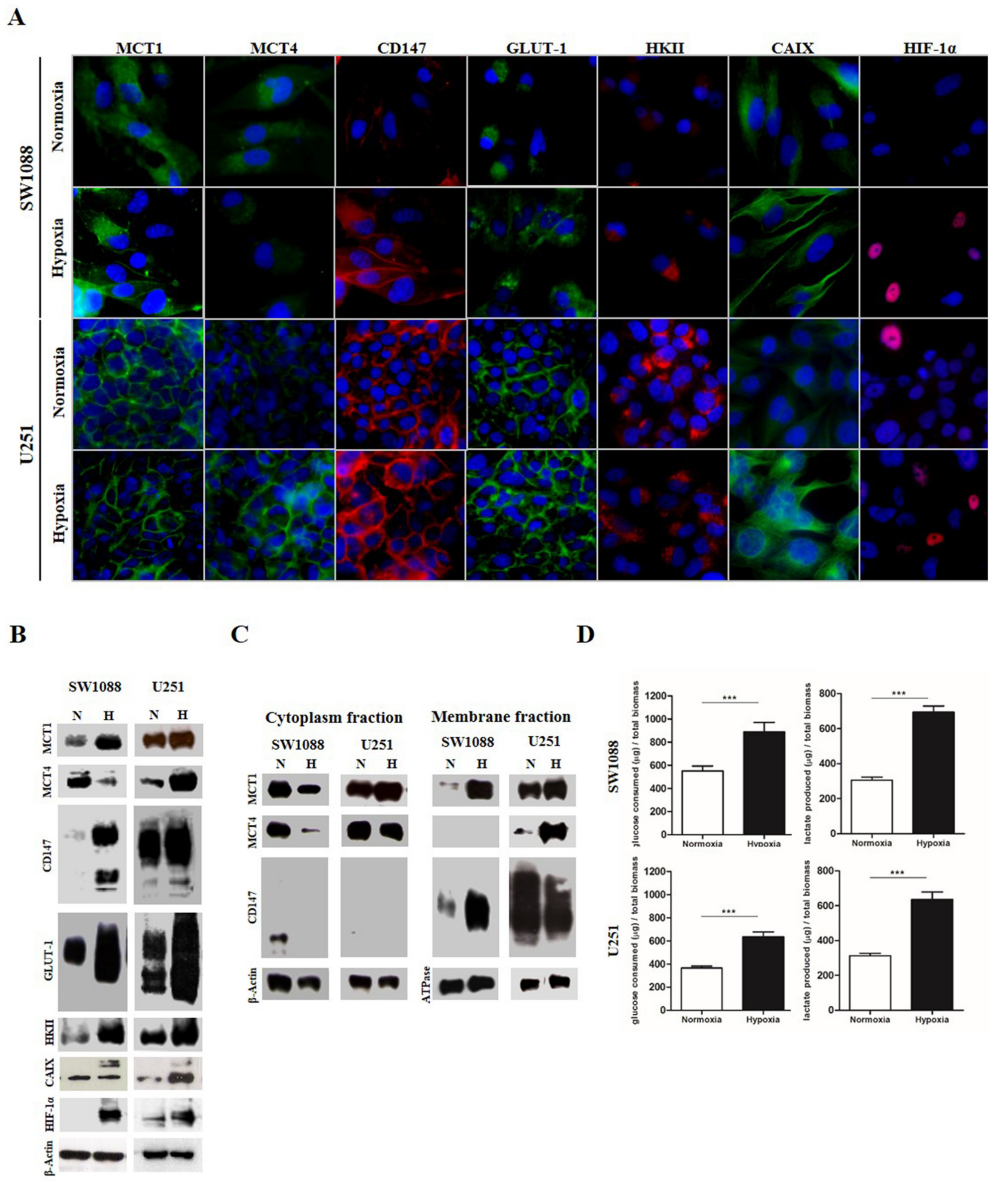
Protein levels, cellular localization and glycolytic metabolism of glioma cells under hypoxia **A.** Cellular localization of the different metabolic markers and monocarboxylate transporters in normoxic and hypoxic regions, by immunofluorescence; pictures were taken using the microscope Olympus BX16 at 400x. **B.** Protein levels under normoxia and hypoxia in glioma cells by Western blot analysis; MCT1 50kDa, MCT4 44kDa, CD147, high glycosylated (HG) 52-42kDa and low glycosylated (LG) 34kDa; GLUT1 52kDa, HKII 95kDa, CAIX 52kDa, HIF-1α 110kDa and β-Actin 42kDa. **C.** Protein levels of cytoplasm and plasma membrane fractions for MCT1, MCT4 and CD147; β-actin and ATPase (90kDa) were used as loading controls for cytoplasm and plasma membrane fractions, respectively. **D.** Glucose consumption and lactate secretion in glioma cells under normoxia and hypoxia conditions; results are the mean±SEM of at least three independent experiments, each one in triplicate; *** p≤0.001 normoxia *vs* hypoxia.

In the present study, we used two known glioma cell lines with distinct metabolic behavior [17]. U251 cells exhibit a more glycolytic phenotype and present high plasma membrane expression of MCT1, CD147 and GLUT-1, as well as cytoplasmic HKII expression compared to SW1088 cells, under normoxia conditions (Figure 1A and 1B). In U251 cells, expression of GLUT-1, HKII and CD147 was similar in both normoxia and induced hypoxia conditions, while there was a clear increase of MCT4 and CAIX in hypoxia (Figure 1A and 1B, Table 1), and a slight increase in MCT1 expression (Figure 1B, Table 1). Regarding SW1088 cells, we observed an increased expression of the metabolic markers, with exception of MCT4 that decreased in hypoxia (Figure 1A and 1B, Table 1). We also observed MCT1, CD147 and GLUT1 increase staining in plasma membrane expression under hypoxia, compared to normoxia (Figure 1A and 1B, Table 1). Hypoxia induced conditions were supported by nuclear expression of the hypoxia marker HIF-1α (Figure 1A and 1B). To confirm the increase in plasma membrane expression of MCT1, MCT4 and their chaperone CD147, cell fractionation with plasma membrane isolation was performed. As observed in Figure 1C, there is an increase in MCT1 and CD147 plasma membrane expression in SW1088 cells, with no MCT4 plasma membrane expression.

**Table 1:** Summary of the results of expression of MCTs and other metabolic markers in hypoxia *vs* normoxia conditions in glioma cell lines

	Hypoxia *vs* Normoxia	
	U251	SW1088
**MCT1**	increased (PM)	increased (PM)
**MCT4**	increased (PM)	decreased (cytoplasm)
**CD147**	similar (PM)	increased (PM)
**GLUT1**	similar (PM)	increased (PM)
**HKII**	similar	increased
**CAIX**	increased	increased

PM- plasma membrane

In U251 cells, the increase in MCT4 plasma membrane expression was evident in hypoxia, whereas there was only a slight increase to MCT1 and no changes in CD147 (Figure 1C). Hypoxia conditions induced up-regulation of glycolytic proteins and MCTs in glioma cells, with higher intensity for the most oxidative cell line SW1088. Subsequently, the extracellular glucose and lactate levels were analyzed to confirm the induction of the glycolytic phenotype. As expected, an increase in glucose consumption and lactate extrusion was observed in both cell lines (Figure 1D). In SW1088 cells, a metabolic switch towards a more glycolytic metabolism was confirmed by the shift ratio extruded lactate/consumed glucose from 0.5 under normoxia to 0.8 under hypoxia. At variance, in the more glycolytic U251 cells the ratio remains close to 1 in both conditions (Figure 1D), despite the hypoxia related increase in glucose consumption and lactate secretion.

### MCT1 mediates lactate efflux in glioma cells

**Figure 2:**
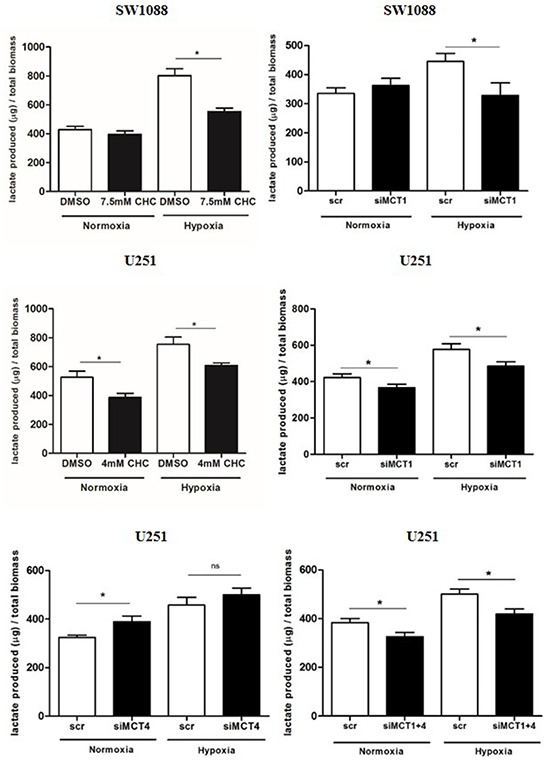
Lactate secretion upon MCT inhibition Extracellular lactate levels upon MCT pharmacological (CHC) and genetic inhibition (siRNA) in SW1088 and U251 cells under hypoxia conditions; results are the mean±SEM of at least three independent experiments, each one in triplicate; *p<0.05 compared CHC or downregulation *vs* control condition.

In order to evaluate the role of MCT1 and 4 as contributors to the glycolytic phenotype in hypoxia, MCT pharmacological inhibition was performed with CHC (IC_50_/2 value) and downregulation of MCT1 and MCT4 isoforms by siRNA. Treatment with either CHC or siMCT1in SW1088 cells led to a decrease in extracellular lactate only in hypoxia (Figure 2). In U251 cells, treatment with CHC decreased extracellular lactate in both normoxia and hypoxia (Figure 2). Similar findings were found for MCT1 downregulation in both conditions, however inhibition of MCT4 alone led to a slight increase in extracellular lactate (Figure 2). Combined downregulation of both MCT isoforms (MCT1 and MCT4) decreased extracellular lactate with a profile similar to MCT1 silencing (Figure 2A). Importantly, specific downregulation of MCT1 had the same effect of CHC on lactate secretion, indicating that MCT1 has an important role in the maintenance of the glycolytic phenotype in hypoxic conditions. Downregulation of MCTs was confirmed by Western blot (Supplementary Figure S1A) and immunofluorescence (Supplementary Figure S1B and S1C). We observed an interdependence of MCT1/CD147, since downregulation of MCT1 led to a decrease in CD147 plasma membrane expression in both cell lines, whereas downregulation of MCT4 in U251 cells did not change the expression and cellular localization of CD147 (Supplementary Figure S1B and S1C).

### MCT1 inhibition decreases glioma cell viability and proliferation under hypoxia

**Figure 3:**
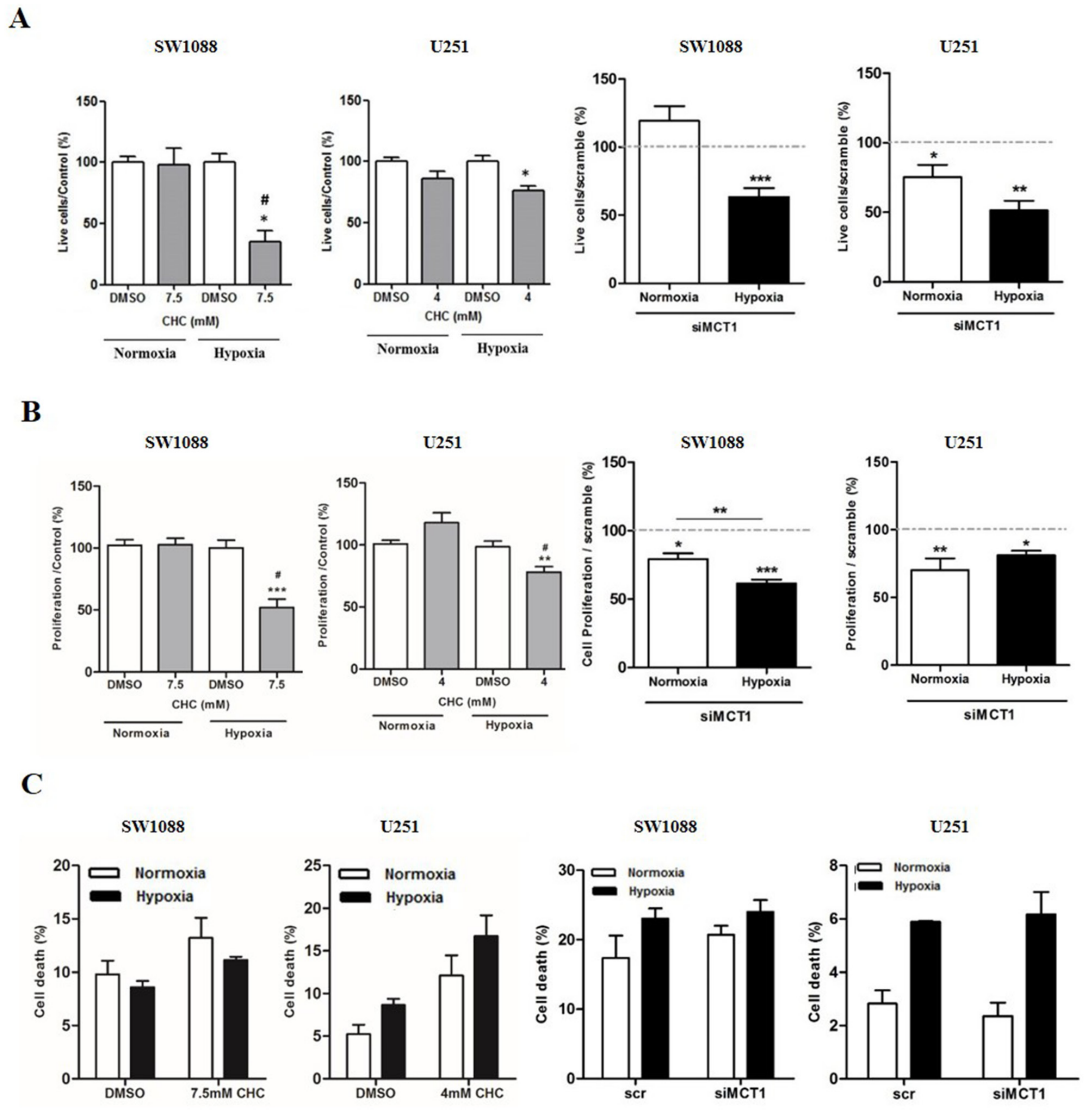
Effect of MCT1 inhibition on cell proliferation and cell death of glioma cells under hypoxia Cell proliferation in SW1088 and U251 cells under hypoxia by **A.** trypan blue and **B.** BrdU assay, respectively. **C.** Cell death in SW1088 and U251 cells under hypoxia by AnnexinV/PI flow cytometry analysis; results are the mean±SEM of at least three independent experiments, each one in triplicate; To MCT downregulation graphs the results were compared to scramble condition which was normalized to 100% represented as grey line; *p<0.05, **p≤0.01 *** p≤0.001; # p<0.05 compared normoxia *vs* hypoxia at respective conditions.

The role of MCT1 on glioma viability and proliferation was also evaluated upon metabolic remodeling induced by hypoxia. Treatment with CHC decreased significantly cell growth in both cell lines under hypoxia (Figure 3A), while only a slight decrease was observed for U251 cells under normoxia. Additionally, downregulation of MCT1 in SW1088 cells, only decreased cell growth under hypoxia (Figure 3A), while in U251 cells, MCT1 silencing led to a significant decrease in cell growth in both normoxia and hypoxia (Figure 3A).

Treatment with CHC only decreased cell proliferation under hypoxia (Figure 3B) and MCT1 downregulation decreased cell proliferation in both cell lines in both normoxia and hypoxic conditions (Figure 3B). Furthermore, it was observed that cell death did not increase in hypoxia either with CHC treatment or MCT1 downregulation, with only a tendency for CHC treatment in U251 cells, but without statistical significance (Figure 3C).

### MCT1 contributes to glioma migration and invasion under hypoxia

The role of MCT1 on cell aggressiveness was evaluated by assessing cell migration and cell invasion upon inhibition of MCT1 activity and expression.

**Figure 4:**
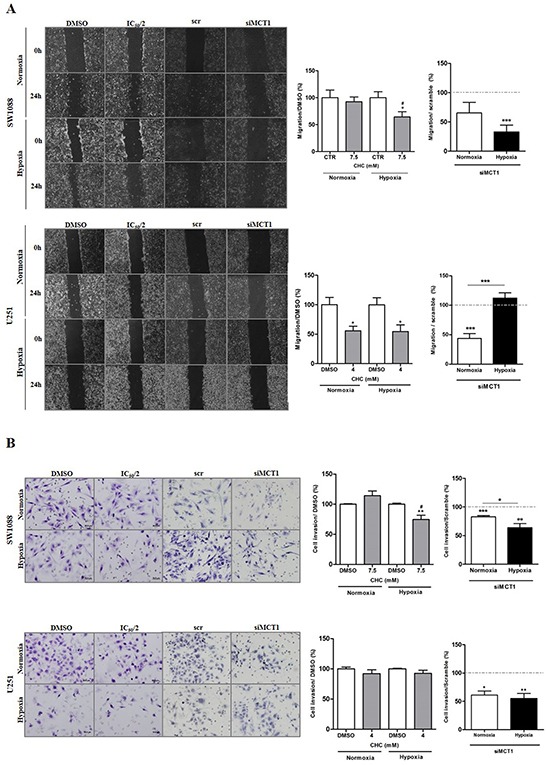
Cell migration and invasion behavior upon MCT1 under hypoxia Cell migration **A.** and cell invasion **B.** of glioma cells under normoxia and hypoxia after MCT1 downregulation and MCT activity inhibition with CHC. Pictures were taken at 40x magnification (migration) and 200x magnification (invasion) in an Olympus BX16 microscope. Results represent the mean±SEM of three independent experiments. The different conditions of silencing were compared to scramble conditions that was normalized to 100% (represented as grey line); *p<0.05, **p≤0.01 *** p≤0.001; # p<0.05 compares normoxia *vs* hypoxia at respective conditions.

In SW1088 cells, treatment with CHC decreased cell migration in both conditions (Figure 4A), with a significant decrease from normoxia to hypoxia. Only treatment with CHC under hypoxia promoted a significant decrease in cell invasion (Figure 4B). Additionally, MCT1 silencing decreased significantly cell migration (Figure 4A) and invasion (Figure 4B) in hypoxia but not under normoxia.

For U251 cells, treatment with CHC decreased significantly cell migration (Figure 4A) but not invasion (Figure 4B) in both normoxia and hypoxia. Downregulation of MCT1 led to a significant decrease in cell migration (Figure 4A) and cell invasion (Figure 4B) under normoxia, however under hypoxia conditions, only a decrease in cell invasion was verified (Figure 4B).

### MCT1 expression increases in hypoxic regions in 3D cultures and in orthotopic glioma models

**Figure 5:**
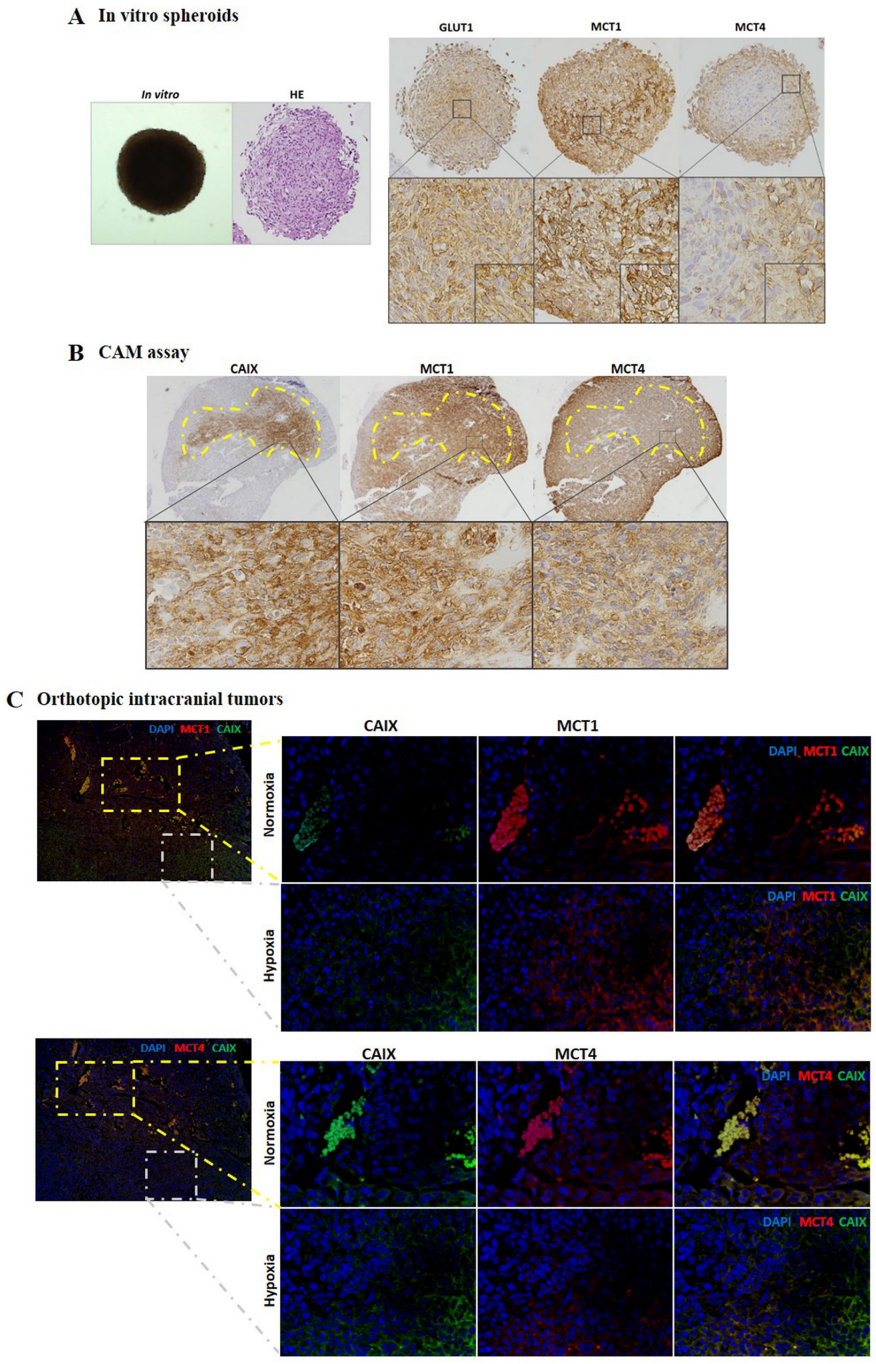
MCT1 and MCT4 expression distribution in 3D culture and in *in vivo* glioma models **A.** Immunohistochemical expression of MCT1, MCT4 and GLUT1 in SW1088 spheroids. Pictures are representative of n=10 spheroids and taken using the Olympus BX16 microscope at 40x, 100x and 400x magnification; **B.** MCT1, MCT4 and CAIX expression in U251 microtumors by immunohistochemistry. Pictures are representative of n=8 eggs and taken using the Olympus BX16 microscope, at 40x, 200x and 400x magnification; **C.** MCT1 and MCT4 expression in *in vivo* U87-MG intracranial tumors by immunofluorescence. Pictures are taken at 100x and highlighted at 400x for MCT1, MCT4 and CAIX in normoxic (close to blood vessels) and hypoxic regions (distant from blood vessels, positive for the hypoxia marker CAIX).

To further support the previous results, we performed SW1088 3D cultures (spheroids), and observed an increase in MCT1 plasma membrane expression in the spheroid core (less oxygenated region), whereas MCT4 expression remained at the periphery and was cytoplasmic (Figure 5A). Additionally, using the CAM assay, we observed in U251 *ex ovo* microtumors that MCT1 plasma membrane expression was higher in the region that was positive for CAIX, while MCT4 presented a weak cytoplasmic expression (Figure 5B). The U87-MG cell line was used in the intracranial model, since is the well described to form *in vivo* tumors that better mimic the human GBM behavior with heterogeneous regions, exhibiting necrotic regions and angiogenic features [29]. Characterization of MCT1 and MCT4 distribution in U87-MG brain tumors showed a weak expression in both isoforms, and a negative CAIX expression, in regions close to blood vessels (normoxic regions) (Figure 5C). Particularly, in the regions positive to CAIX expression (hypoxic regions), we observed a strong co-localization of MCT1 with CAIX (Figure 5C). In contrast, most CAIX positive cells did not co-localize with MCT4 in these intracranial tumors (Figure 5C).

### MCT1 plasma membrane expression is associated with hypoxic regions of GBM tissues

**Figure 6:**
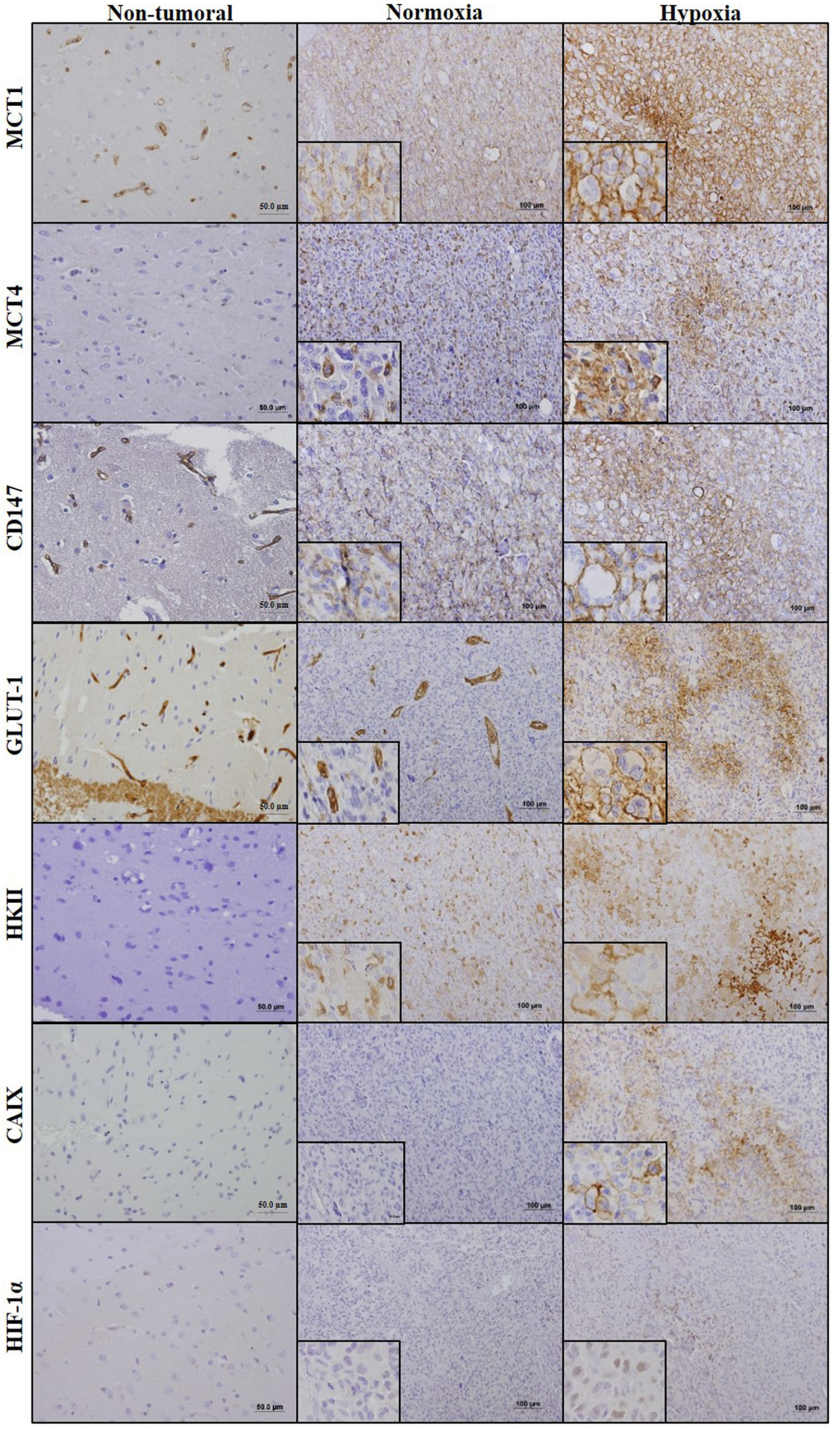
Immunohistochemical expression of monocarboxylate transporters, CD147, GLUT1, HKII, CAIX and HIF-1α in GBM and normal adjacent tissues Expression of MCT1, MCT4, CD147 (MCT1/4 chaperone), glycolytic markers (HKII, GLUT1) and hypoxia markers (CAIX, HIF-1α) in neoplastic and non-neoplastic of GBM patient tissues, by immunohistochemistry. Pictures were obtained using the Olympus BX61 microscope, at 100x and the inserts at 400x magnification.

To support the above finding, a total of 45 GBM cases were assessed for MCT1, MCT4 and chaperone CD147 expressions, as well as for different glycolytic metabolism-related proteins, GLUT-1, HKII, CAIX and HIF-1α in normoxic regions (vascularized regions, distant from necrotic areas) and hypoxic regions (peri-necrotic areas). The expression of these markers was also evaluated in 17 non-tumoral adjacent tissues. In the non-tumoral regions, astrocytes and neurons were negative for all markers (Figure 6), and expression of MCT1, CD147 and GLUT-1 was present in capillaries and small vessels (Figure 6). In GBM tissues, CAIX, GLUT-1 and HIF-1α presented a focal expression in hypoxic regions, with 88.9%, 88.9% and 60% positivity, respectively (Table 2; Figure 6). MCT1 total (cytoplasm/membrane) expression increased in hypoxic regions of GBMs (62.2%) (Table 2; Figure 6), with 71.1% of cases presenting plasma membrane expression (Table 2; Figure 6). MCT4 and CD147 were expressed in both normoxic and hypoxic regions (82.2% and 91.1%, respectively; Table 2, Figure 5), however only 20% of the cases presented MCT4 plasma membrane expression in hypoxic regions (Table 2). The MCT chaperone CD147 exhibited a higher expression at the plasma membrane, with 37.8% of cases positive only in hypoxic regions (Figure 6; Table 2). The glycolytic marker HKII presented a ubiquitous expression with 44.4% of total cases positive only in hypoxia (Table 2; Figure 6).

**Table 2:** Distribution of metabolic markers and MCT/CD147 expression in normoxic and hypoxic regions of glioblastoma tissues

	n (total)	Total expression				Plasma membrane expression			
		Hypoxia		Normoxia and Hypoxia		Hypoxia		Normoxia and Hypoxia	
		n	(%)	n	(%)	n	(%)	n	(%)
**MCT1**	45	28	62.2	17	37.8	32	71.1	8	17.8
**MCT4**	45	8	17.8	37	82.2	9	20.0	10	22.2
**CD147**	45	4	8.9	41	91.1	17	37.8	26	57.8
**GLUT-1**	45	40	88.9	4	8.9	39	86.7	2	4.4
**CAIX**	45	40	88.9	2	4.4	32	71.1	0	0.0
**HKII**	45	20	44.4	25	55.6	na*	-	na*	-
**HIF-1α**	45	27	60.0	0	0.0	na*	-	na*	-

* na: not applicable

**Figure 7:**
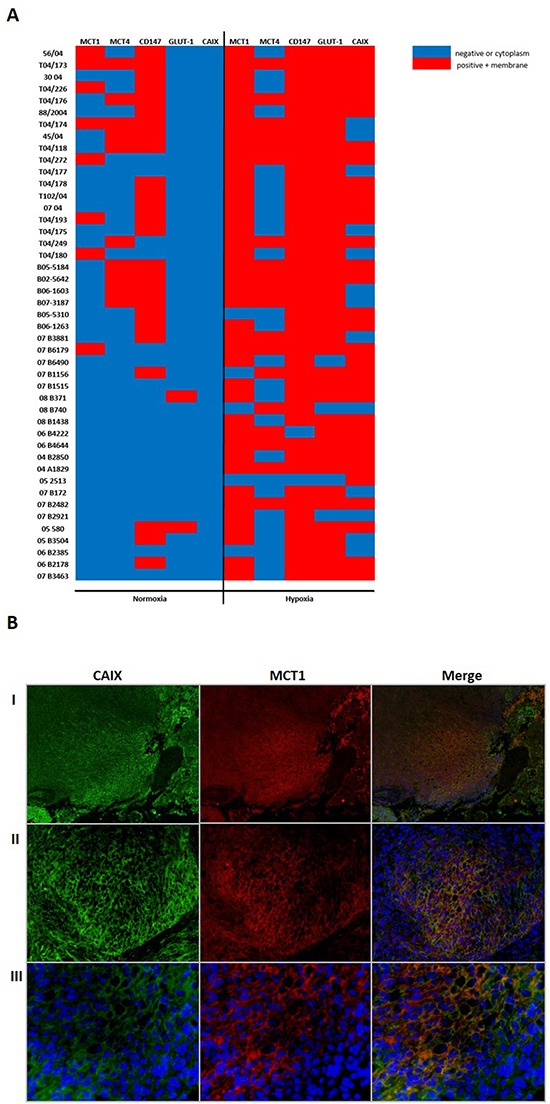
Association of MCTs and CD147 expression with hypoxia markers (GLUT-1 and CAIX) in GBMs **A.** Heat map representation of the monocarboxylate transporter and chaperone expression, and hypoxic markers for the 45 GBM tissues evaluated under normoxia and hypoxia regions. Blue color corresponds to negative or cytoplasm expression and red corresponds to plasma membrane expression; **B.** Plasma membrane expression of the hypoxia marker CAIX (in green) and MCT1 (in red) in GBM tissue. Co-localization of MCT1 and CAIX at the plasma membrane (orange). Pictures were taken using the microscope Olympus BX16 at 100x (I), 200x (II) and 400x (III).

In the immunostaining heat map (Figure 7A), we observed that MCT1 expression increases from normoxic to hypoxic regions, showing higher plasma membrane expression in hypoxic regions together with the hypoxic (CAIX) and glycolytic (GLUT-1) markers. We verified that the cases with positivity in normoxic regions maintain the expression in hypoxic regions. Additionally, we found an association of MCT1 plasma membrane expression in hypoxic regions of GBM with co-expression of HIF-1α and CAIX, and also a tendency to associate with GLUT-1 (Table 3). Moreover, co-localization of MCT1 with CAIX at the plasma membrane in hypoxic regions of the GBM tissues was confirmed by immunofluorescence (Figure 7B). Importantly, clinical specimens of GBMs validated the previous findings using *in vitro* and *in vivo* GBM models, in which MCT1 is enriched in hypoxic regions, and co-localize with surrogate markers of hypoxia.

**Table 3:** Association of MCT1 plasma membrane expression in hypoxic regions with different hypoxia-inducible metabolic markers in glioblastomas

		MCT1			
		n	PM*	(%)	*p*
**MCT4**	negative/cytoplasm	36	27	60.0	0.250
	positive/PM	9	5	11.1	
**CD147**	negative/cytoplasm	28	20	44.4	0.952
	positive/PM	17	12	26.7	
**GLUT-1**	negative/cytoplasm	6	4	8.9	0.067
	positive/PM	39	28	62.2	
**CAIX**	negative/cytoplasm	13	9	20.0	**0.031**
	positive/PM	32	23	51.5	
**HKII**	negative	20	20	44.4	0.141
	positive	25	12	26.7	
**HIF-1α**	negative	18	16	35.6	**0.032**
	positive	27	16	35.6	

* positivity for MCT1 plasma membrane (PM) in hypoxic regions of glioblastomas

## DISCUSSION

Glycolytic metabolism of tumor cells has been described as an adaptive mechanism to intermittent hypoxia present in pre-malignant lesions [4, 6]. Yet, regulation of MCTs in hypoxia is not well understood, with controversial results, namely for MCT1 [20-22, 26, 27]. GBM are characterized by the presence of necrosis that seems to be associated with hypoxia regions [30], and these high levels of hypoxia have been implicated in resistance to chemo- and radiotherapy [31]. In a previous study, we reported up-regulation of MCT1, MCT4 and CD147 in a series of GBM compared to normal tissues [17]. Herein, we intended to understand the role of hypoxia in the regulation of MCT expression and activity in GBM.

**Figure 8:**
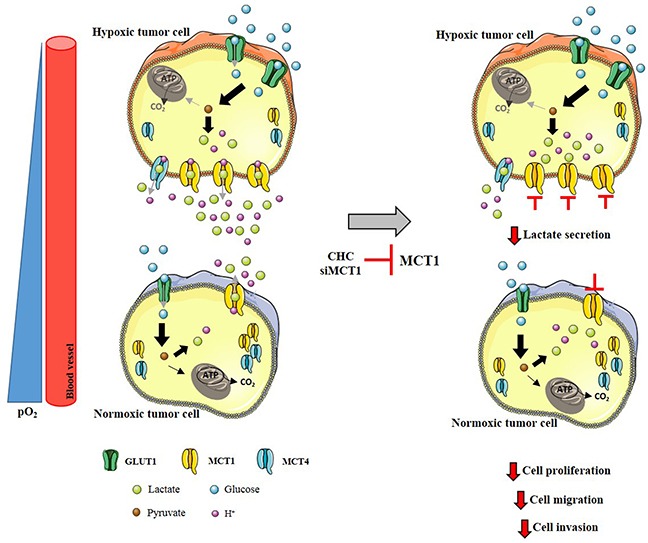
MCT1 as a mediator of aggressiveness at hypoxic regions in glioblastomas In normoxic tumor cells, MCT1 is expressed at the plasma membrane, whereas MCT4 remains at the cytoplasm. Hypoxia induces upregulation of MCT1 plasma membrane expression, which is responsible for lactate efflux. Inhibition of MCT1 expression or activity impairs lactate secretion, contributing to a decrease in cell proliferation, cell migration and invasion.

Analyzing two cell lines with different metabolic phenotypes, namely a less glycolytic SW1088 cell line (lower MCT1 plasma membrane expression) and a more glycolytic U251 cell line (higher MCT1 plasma membrane expression), we observed distinct sensitivities to the classical pharmacological MCT inhibitor CHC [17]. In the present study, we showed that hypoxia led to MCT1 and CD147 up-regulation, but not MCT4, at the cell plasma membrane of SW1088 cells. However, there is compelling evidence in the literature demonstrating that MCT4 is the isoform which is upregulated in the hypoxic regions of the tumors, being responsible for lactate efflux to the tumor microenvironment, being associated with several malignant features [20-23, 27]. Upregulation of MCT4 levels in hypoxia was observed in bladder cancer [20] and an increase in MCT4 plasma membrane expression was also observed in breast cancer cells [22]. Importantly, Ullah *et al.* reported that only MCT4 is induced by hypoxia, since only this MCT isoform contains hypoxia response elements (HRE) for HIF-1α [27]. At variance with these authors, we showed that MCT4 expression decreased with hypoxia in SW1088 cells. Accordingly, it was described that MCT4 expression decreased after chronic hypoxia in the plantaris muscle [24]. Moreover, a recent study in adipocytes demonstrated that MCT1, but not MCT4 protein expression increased in hypoxia [26]. Here, a slight increase in MCT1 was observed in hypoxia in the highly glycolytic cell line (U251), however an increase in MCT4 plasma membrane expression was also verified, supporting the study of Ullah *et al.,* [27]. Additionally, an increased MCT1 and MCT4 plasma membrane density was reported in brain cells under hypoxia [22], which also corroborates our results (Figure 8).

Up-regulation of MCT1 contributes to the glycolytic phenotype of brain cancer cells, which was supported by the observed increase in glucose consumption and lactate production, as well as overexpression of GLUT1 and HKII in SW1088 cells in hypoxia. In the present work, we also investigated the effect of MCT activity inhibition in human glioma cell lines in hypoxia, using the lactate transport inhibitor CHC and also through MCT1 and MCT4 mRNA downregulation (siRNA). MCT1 inhibition induced a decrease in lactate production in SW1088 cells in hypoxia compared to normoxia conditions. This was anticipated, since hypoxia induces the glycolytic phenotype in SW1088 cells, leading to increased MCT1 plasma membrane expression. Additionally, MCT1 silencing in U251, but not MCT4, decreased lactate production in both normoxia and hypoxic conditions. Although previous reports suggested that MCT4 is the major lactate transporter in hypoxic cells [21, 27], in our cell models we demonstrate that MCT1 mediates the lactate efflux in hypoxic conditions. In line with this, a recent study with colon adenocarcinoma cells showed that MCT1 silencing in hypoxia decreased lactate efflux [32]. Furthermore, our results show that CHC inhibited preferentially MCT1 rather than MCT4 isoform, which can be explained by the different Ki values for these two isoforms [33, 34].

It is known that hypoxia contributes to the glycolytic-acidic resistant phenotype of tumor cells [6], contributing to increased cell growth and survival, due to induction of several malignant features like migration and invasion. Accordingly, we studied the contribution of MCTs, particularly MCT1, to cell growth, survival and invasiveness capacity under hypoxia (Figure 8). Inhibition of MCT1 activity and expression led to a decrease in viability and cell proliferation, however it did not promote cell death in hypoxia-induced SW1088 cells. In fact, some studies have been demonstrating the importance of lactate transport via MCT1 in the promotion of cell proliferation and tumor growth [35, 36].

GBM have an intrinsic high invasive capacity [37], producing high amounts of lactate [12] that contribute to the acidic microenvironment, and consequent promotion of cell migration and invasion. It is already described that inhibition of MCT activity decreases breast cancer cell migration [36] and the invasive capacity of GBM [38]. Additionally, it was described that MCT1 is the mediator of lactate efflux and consequently promotes the invasive phenotype of GBM [17]. However, the role of MCTs in the promotion of migration and invasion under hypoxia was not assessed. In the present study, MCT1 downregulation in hypoxia was associated with a decrease in cell invasion in both cell lines tested. However, a decrease in cell migration was only observed in SW1088 cells. Since MCT4 does not appear to participate in lactate efflux even at hypoxic conditions in our cell model, the increase in cell migration after MCT1 downregulation could be explained due to the strong association of MCT4 with β1-integrin on the cell movement capacity [39]. Additionally, the effect observed can be a mechanism mediated by hypoxia, since it is known that under these conditions there is up-regulation of several proteins associated with cell migration and invasion [40]. Furthermore, lactate transport in cancer cells could be mediated by a different lactate transporter, namely the sodium monocarboxylate transporter 1 (SMCT1) [41]. Although its expression is not well described in tumors, we cannot exclude the possible up-regulation of SMCT1 in hypoxia as a compensatory mechanism for cell survival. Regardless, our study demonstrates that MCT1 is a mediator of the aggressiveness phenotype of GBM cells in hypoxia, since there was a decrease in cell invasion after MCT1 downregulation (Figure 8).

Based on our findings, we hypothesize that up-regulation of MCT1 in GBM cells can be an adaptation to changes in oxygen levels, helping cells to be prepared for episodes of net release of lactate, alternating with episodes of net uptake. In fact, our results with 3D cultures (SW1088 spheroids), *ex ovo* U251 microtumors and U87-MG brain tumors support the role of MCT1 as mediator of lactate efflux under low oxygen level conditions. Although, our results showed ubiquitous expression of MCT1 in SW1088 spheroids and *in vivo* glioma models, MCT1 plasma membrane expression is stronger in hypoxic regions and co-localizes with CAIX expression *in vivo* glioma models.

Furthermore, the importance of enhanced MCT1 expression in hypoxia as a gatekeeper of lactate efflux was confirmed in a series of 45 GBM patients. The present study demonstrates that there is a shift from cytoplasm to plasma membrane expression of MCT1 in GBM tissues, while MCT4 is present in higher amounts in the cytoplasm, despite the increase in both MCT1 and MCT4 expression in hypoxia (Figure 8). Additionally, MCT1 plasma membrane expression was associated with positivity for HIF-1α and CAIX in hypoxic regions. A previous report in rat brain tumors described that MCT4 expression was located in the center of the tumor (hypoxic area), while MCT1 was mainly at the periphery [42]. On the other hand, Kaanders *et al.*, demonstrated that MCT1 was present in hypoxic regions in head and neck tumors and was associated with CAIX expression [43]. Although MCT4 is the isoform that is described to be induced by hypoxia [27], a recent study by Feron *et al.* indicates that hypoxia also induced an increase in MCT1 mRNA levels in breast cancer [32]. Additionally, hypoxia induced MCT1 protein expression, but not MCT4, in human adipocytes [26]. Altogether, these findings suggest that besides MCT4, MCT1 expression is also regulated by hypoxia, likely supporting the maintenance of the glycolytic phenotype of GBM by mediating lactate efflux (Figure 8).

To support our results, we studied in parallel the expression of glycolytic markers (GLUT-1 and HKII) and hypoxia markers (CAIX and HIF-1α), and observed that CAIX, HIF-1 and GLUT-1 expression was restricted to hypoxic regions. These results are supported by previous studies reporting that HIF-1α [44], GLUT-1 [45] and CAIX [46] expression was increased in GBM compared to low grade gliomas and that they are associated with necrotic regions (hypoxic regions). In the present study, MCT1 plasma membrane expression was associated with HIF-1α and CAIX in human GBM tissues and a similar pattern was observed in CAIX in the CAM U251 microtumors and U87-MG intracranial mice brain tumors, which supports the association of MCT1 expression with hypoxic tumors regions. In accordance to our model, a study by Pinheiro *et al* also showed that MCT1 was associated to CAIX expression in breast cancer tissues [47]. On the other hand, and as expected, several studies reported association of MCT4 with hypoxia markers in different solid tumors [20, 43, 48]. In head and neck [43], and lung cancer [48] higher MCT4 expression was observed in regions distant from blood vessels and was associated with the hypoxia markers pimonidazole and GLUT1, respectively. In the whole, our results support that MCT1 expression is regulated by hypoxia, not directly through HIF-1α but perhaps through a mechanism that could be dependent on HIF-1α activation.

## CONCLUSIONS

MCT1 plasma membrane expression is upregulated in hypoxia in GBM models and human tissues, playing an important role in the maintenance of their glycolytic phenotype, which in turn is associated with higher aggressiveness. Thus, targeting MCT1, alone or in combination with conventional therapy, can constitute an attractive strategy for GBM therapy.

## MATERIALS AND METHODS

### Cell culture and cell lines

U251, SW1088 and U87-MG cell lines used in this study were grown in standard conditions [17]. U251 cells were kindly provided by Professor Joseph Costello, California University, Neurosurgery Department, San Francisco, USA. SW1088 and U87-MG cells were obtained from ATCC (American Type Culture Collection). Cell line authentication was performed by IdentiCell Laboratories (Department of Molecular Medicine (MOMA) at Aarhus University Hospital Skejby in Åarhus, Denmark). Genotyping confirmed the complete identity of cell lines.

### Hypoxia induction

Induction of hypoxia was performed using hypoxic chambers (Modular Incubator Chamber (MIC-101), billups-rothenberg.inc). Cells were placed in an airtight chamber with hypoxic gas mixture 0% O_2_, 5% CO_2_ and 95% N_2_. The hypoxic chamber was then placed in an incubator at 37°C for a specific period time, according to the assay to be carried out. The O_2_ concentration during assays was controlled by an O_2_ sensor, being below 1% throughout the experiments. The control normoxic conditions were performed in a humidified incubator at 21% O_2_, 5% CO_2_ and 74% N_2_ for the same period of time.

### Antibodies

Antibody details and conditions for immunohistochemical, immunofluorescence and Western blot assays are summarized in Table 4.

**Table 4:** Details about primary antibodies and conditions used in immunohistochemistry, immunofluorescence and western blot

Protein	Company	Dilution	Clone
MCT1	Chemicon International (AB3538P)	1:200	2424624
MCT4	Santa Cruz Biotechnology (sc-50329)	1:500	C1915
CD147	Santa Cruz Biotechnology (sc-71038)	1:500	F1512
GLUT-1	AbCam (ab15309)	1:500	GR207686-2
CAIX	AbCam (ab15086)	1:2000	GR154000-1
HKII	AbCam (ab104836)	1:750	GR164816-6
HIF-1α	BD Biosciences (610958)	1:100	4115908
β-Actin	Santa Cruz Biotechnology (sc-1616)	1:300	I0709

### Downregulation of MCTs

Downregulation of MCT1 or MCT4 was performed with siRNA (siRNA for MCT1, s580, Ambion; scramble siRNA, 4390843, Ambion, siRNA for MCT4, S17417) using lipofectamine as a transfection reagent, according to the manufacturer's instructions [49].

### Immunohistochemistry

Representative 4μm-thick tissue sections were used for immunohistochemical analysis. Immunohistochemistry (IHC) for MCT1, CD147 and HIF-1α was performed according with the avidin-biotin-peroxidase technique (R.T.U. Vectastain Elite ABC kit; Vector Laboratories), as previously described [17]. The Ultravision Detection System Anti-polyvalent, HRP (Lab Vision Corporation) was used for MCT4, GLUT-1, CAIX and HKII, IHC, as previously described [17].

Briefly, deparaffinised and rehydrated slides were submitted to adequate heat-induced antigen retrieval for 20min at 98°C with 10mM citrate buffer (pH 6.0) for MCT1, MCT4, GLUT-1 and CAIX and 1mM EDTA buffer (pH 8.0) for CD147, HKII and HIF-1α. After endogenous peroxidase inactivation, incubation with the primary antibodies was performed overnight for MCT1, CD147 and HIF-1α and 2h for MCT4, GLUT-1, CAIX and HKII, at room temperature. The immune reactions were visualized with 3,3′-diaminobenzidine (DAB+ Substrate System; Dako) as a chromogen. Tissue immunostaining was evaluated by an experienced neuropathologist (MH). Analysis was performed semi-quantitatively considering the intensity of staining, according to the following score: score 0: negative or weak staining and score 1 for moderate and strong staining. Score 0 was defined as negative and score 1 as positive. The intensity of staining was evaluated in regions close to blood vessels, here defined as normoxic regions, and, in regions close to necrosis, defined as hypoxic regions. Cellular localization of staining (cytoplasm/membrane) was also evaluated.

### Immunofluorescence

U251 and SW1088 cells were seeded on cover slips at a density of 30.000 cells/well, overnight. Briefly, after 24h in hypoxia, cells were fixed and permeabilized in methanol during 20min. After blocking with 5% bovine serum albumin (BSA) for 30min, cells were incubated overnight at room temperature with the primary polyclonal antibodies. Then, 1h of incubation with the secondary antibody anti-rabbit-Alexa Fluor 488 (1:500 dilution, A11008, Invitrogen) in 5% BSA was performed for MCT4, MCT1, GLUT-1 and CAIX and anti-mouse-Alexa Fluor 594 antibody (1:250 dilution, A11032, Invitrogen) for HKII, CD147 and HIF-1α. Finally, after washing in PBS, cells were mounted in Vectashield Mounting Media with 4′,6-diamidino-2-phenylindole (DAPI) (Vector Laboratories) and images were obtained with a fluorescence microscope (Olympus IX81), using the Cell P software. The procedure for GBM tissues was similar, performing the antigen retrieval before the blocking with 10% BSA for 45min.

### Western blotting

Western blot was performed as described previously [17]. Incubation with primary antibodies (Table 4) was performed overnight at 4°C. Bound antibodies were visualized by chemiluminescence (Supersignal West Femto kit; Pierce). β-Actin was used as loading control.

For subcellular fractionation, cells were washed with cold PBS and then incubated with 0.5mg/ml of Sulfo-NHS-LC- Biotin in PBS (3ml/T75 flask) during 30min at 4°C. Cells were then washed 3x with cold PBS 10mM Glycine, followed by cellular lysis. Then, 400μg of protein was incubated with 50μl of Streptavidin Agarose Resin beads in PBS, overnight with shaking at 4°C. Proteins were centrifuged at 6000rpm for 1min and the supernatant (cytoplasm fraction) was collected and quantified. The bead pellet (membrane fraction) was washed 4x in 1ml lysis buffer, added to 60μl loading buffer and stored at −80°C, until use. For Western blot analysis, cells were previously exposed to hypoxia for 24h.

### Extracellular glucose and lactate measurements

Cells were plated in 48 well plates at a density of 3×10^4^ cells per well and allowed to adhere overnight. Glucose and lactate content was analyzed in cell culture medium after 24h of hypoxia stimulation in treated IC_50_/2 CHC value and MCT downregulated cells, using commercial kits (Roche and Spinreact, respectively), as described [17]. For these time points, the total protein (expressed as total biomass) was assessed using the sulforhodamine B assay (SRB, TOX-6, Sigma-Aldrich) [17]. Results are expressed as total μg/total biomass.

### Cell viability and proliferation assays

U251 and SW1088 cells were plated in 24 well plates, at density of 3×10^4^ cells/well and allowed to adhere overnight. After 24h of stimulation in hypoxia, cells were treated with the IC_50_/2 values of CHC (4mM in U251 and 7.5mM in SW1088 cells, respectively) (Alpha-cyano-4-hydroxycinnamate, Sigma-Aldrich) in DMEM without FBS, under normoxia and hypoxic conditions, during 48h. After treatment, cells were collected and the effect of CHC on cell viability was assessed through trypan blue counting. For cell proliferation analysis, cells were plated into 96-well plates, at a density of 3×10^3^ cells per well and treated with CHC using the same conditions that described for trypan blue assay. After CHC treatment, cells were incubated with BrdU and incorporation was assessed at 450nm (λ_ref_=655nm), according to the manufacturer's protocol (BrdU, Cell Proliferation ELISA; Roche Diagnostics). Cell viability (trypan blue assay) and cell proliferation for downregulated MCT1 glioma cells, were performed as described above.

### Wound-healing assay

Cells were seeded in 6-well plates and cultured to at least 95% confluence and wound-healing assay was performed as described previously [17]. Treatment with IC_50_/2 value of CHC and MCT1 downregulation were performed during 24h in hypoxia. Wound areas were photographed at 0 and 24 hours.

### Invasion assay

Cell invasion of U251 and SW1088 cells was assessed using 24 well BD Biocoat Matrigel Invasion Chambers (354480, BD Biosciences), as previously described [17, 50]. Before seeding on rehydratated Matrigel Matrix Chambers, cells were grown in 6-well plates in hypoxia and normoxia conditions during 24 hours. After that, U251 and SW1088 cells, at a density of 2.5×10^4^ cell per well in DMEM without FBS plus CHC treatment (IC_50_/2 values) were added to each insert. The same procedure was followed for MCT1 silenced cells. After 24hours, non-invading cells were removed and invading cells were fixed with methanol and stained with hematoxylin.

### Spheroid formation

To form spheroids, 2×10^3^ SW1088 cells were seeded in agarose-coated 48-well plates in 200μl of DMEM supplemented media. Spheroids were incubated, under shaking, at 37°C in a humidified atmosphere of 5% CO_2_ for 11 days. Fresh culture media was added every 48 hours. At day 15, intact spheroids were collected, fixed with 4% paraformaldehyde and embedded in paraffin for IHC analyses, as previously described.

### Chicken chorioallantoic membrane (CAM) assay

CAM assay was performed as described previously [17, 51]. U251 cells (2×10^6^ cells) in Matrigel were placed at 10 day of embryo development on the CAM and allowed to growth for 7 days. After that, CAMs with tumors were dissected, fixed in 4% paraformaldehyde at room temperature and included in paraffin. Immunohistochemistry for MCT1, MCT4 and CAIX on paraffin sections of microtumors was performed as previously described for human samples.

### Orthotopic GBM xenografts

All experiments with mice were approved by institutional and national ethical committees (Direção Geral de Alimentação e Veterinária, Portugal) and in accordance with European Union Directive 2010/63/EU. For intracranial models, a total of 2×10^5^ U87-MG cells were stereotactically injected in the brain striatum (1.8mm medial-lateral right, 0.4mm anterior-posterior, and 2.5mm dorsal-ventral from the bregma) of 8 weeks old athymic nude Foxn1 *^nu^* male mice, as previously described [52]. Animal body weight was evaluated 3 times per week, and general behavior and symptomatology daily. Brains were collected for immunofluorescence analyses, for which samples were fixed by immersion in formalin and subsequently embedded in paraffin.

### Tissues samples

A total of 45 formalin-fixed paraffin embedded (FFPE) GBM (WHO grade IV) and 17 non-tumoral adjacent tissues were obtained from the Department of Pathology of Hospital Pedro Hispano, Matosinhos, Centro Hospitalar do Porto, Porto, and Hospital Dr. Nélio Mendonça, Madeira, Portugal. All procedures described in this study were in accordance with national and institutional ethical standards and previously approved by Local Ethical Review Committees.

### Statistical analysis

Data from human tissues were analyzed using SPSS statistical software (version 22, SPSS Inc). Comparison of expression of the different markers was evaluated for statistical significance using Pearson's chi square (χ^2^) test with the threshold for significance being *p*≤0.05. For *the in vitro* studies, the GraphPad prism 5 software was used, with the Student's *t* test, considering significant values *p*≤0.05.

## SUPPLEMENTARY FIGURE


