# Supplementary material for: Hypoxia-mediated upregulation of MCT1 expression supports the glycolytic phenotype of glioblastomas
Source: Oncotarget. 2016 Jun 16;7(29):46335–53. doi: 10.18632/oncotarget.10114 (PMC5216802; doi:10.18632/oncotarget.10114)
Supplement: Supplementary file 1 [file oncotarget-07-46335-s001.pdf]

Hypoxia-mediated upregulation of MCT1 expression supports the glycolytic phenotype of glioblastomas

SUPPLEMENTARY FIGURE

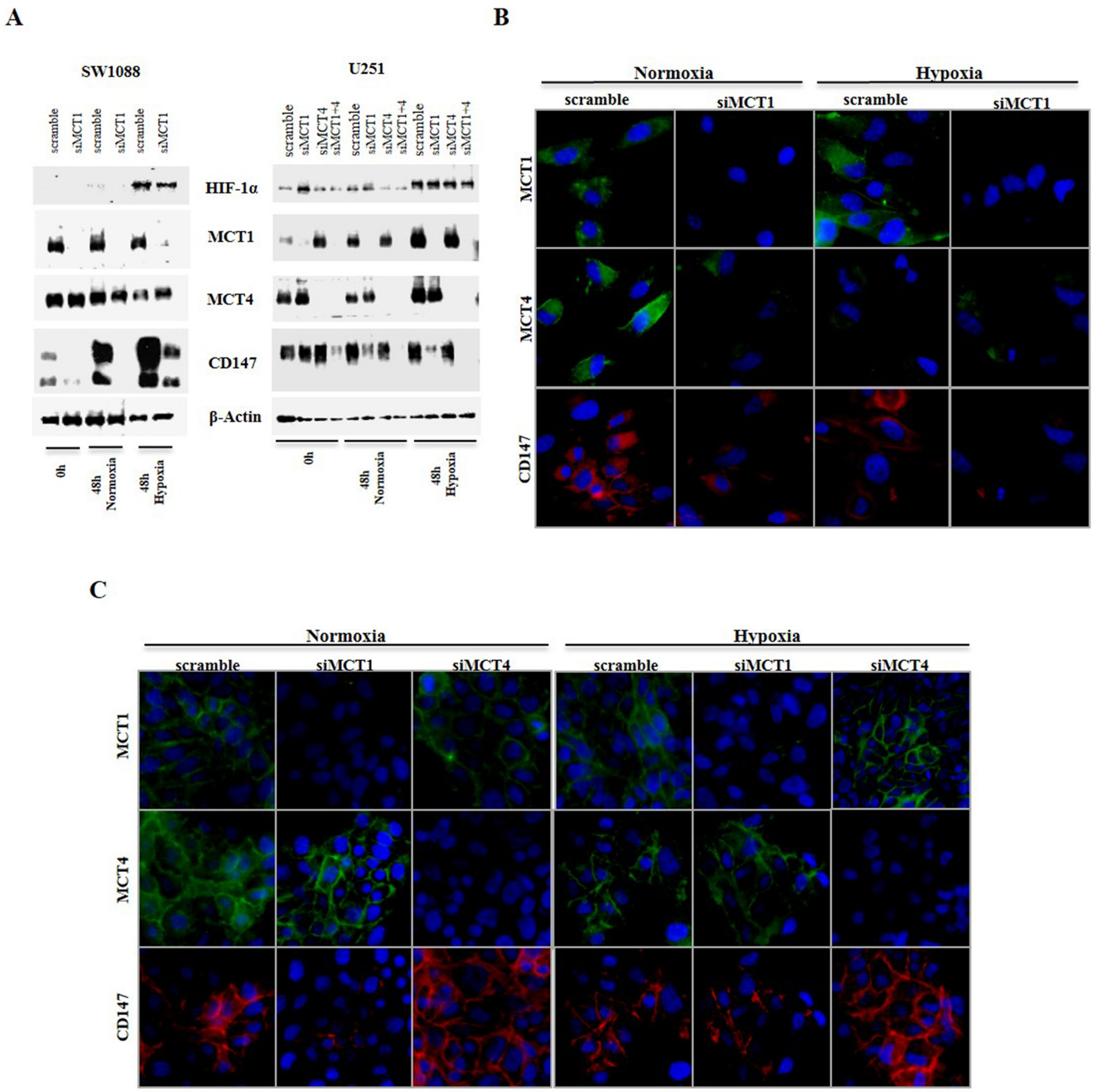

Supplementary Figure S1: MCTs and chaperone CD147 expression in silenced glioma cells. A. MCT1, MCT4, CD147 and HIF-1α expression after MCT isoform silencing in U251 and SW1088 cells (Western blot) MCT1 50kDa, MCT4 44kDa, CD147, high glycosylated (HG) 52-42kDa and low glycosylated (LG) 34kDa; HIF-1α 110kDa and β-Actin 42kDa. MCT1, MCT4 and CD147 expression after MCT silencing in **B.** SW1088 and **C.** U251 cells (immunofluorescence). MCT1 downregulation decreases CD147 expression under both normoxia and hypoxia conditions.
